# Efficacy and safety of deep brain stimulation in mesencephalic locomotor region for motor function in patients with post-stroke hemiplegia: a study protocol for a multi-center double-blind crossover randomized controlled trial

**DOI:** 10.3389/fneur.2024.1355104

**Published:** 2024-08-13

**Authors:** Junpeng Xu, Bin Liu, Shuzhen Liu, Zhebin Feng, Yanyang Zhang, Di Liu, Qing Chang, Haonan Yang, Yuhan Chen, Xinguang Yu, Zhiqi Mao

**Affiliations:** ^1^Medical School of Chinese PLA, Beijing, China; ^2^Department of Neurosurgery, The First Medical Center of Chinese PLA General Hospital, Beijing, China; ^3^Department of Chengde Medical University, Chengde, China; ^4^The First Clinical Medical College of Hebei North University, Zhangjiakou, China

**Keywords:** deep brain stimulation, hemiplegia, motor dysfunction, motor recovery, stroke

## Abstract

**Background:**

Deep brain stimulation (DBS) is a potential treatment for improving movement disorder. However, few large-sample studies can reveal its efficacy and safety. This study aims to initially explore the efficacy and safety of DBS in the mesencephalic locomotor region (MLR) on motor function in patients with post-stroke hemiplegia.

**Methods/design:**

This multicenter, prospective, double-blind, randomized crossover clinical trial aims to assess the safety and effectiveness of Deep Brain Stimulation (DBS) in the mesencephalic locomotor region (MLR) for patients with moderate to severe post-stroke hemiplegia. Sixty-two patients with stable disease after a year of conservative treatment will be enrolled and implanted with deep brain electrodes. Post-surgery, patients will be randomly assigned to either the DBS group or the control group, with 31 patients in each. The DBS group will receive electrical stimulation 1 month later, while the control group will undergo sham stimulation. Stimulation will be discontinued after 3 and 6 months, followed by a 2-week washout period. Subsequently, the control group will receive electrical stimulation, while the DBS group will undergo sham stimulation. Both groups will resume electrical stimulation at the 9th and 12th-month follow-ups. Post-12-month follow-up, motor-related scores will be collected for analysis, with the Fugl-Meyer Assessment Upper Extremity Scale (FMA-UE) as the primary metric. Secondary outcomes include balance function, neuropsychiatric behavior, fall risk, daily living activities, and quality of life. This study aims to provide insights into the therapeutic benefits of DBS for post-stroke hemiplegia patients.

**Result/conclusion:**

We proposed this study for the first time to comprehensively explore the effectiveness and safety of DBS in improving motor function for post-stroke hemiplegia, and provide evidence for DBS in the treatment of post-stroke hemiplegia. Study limitations are related to the small sample size and short study period.

**Clinical Trial Registration:**

Clinicaltrials.gov, identifier NCT05968248.

## Introduction

The number of stroke patients worldwide has exceeded 20 million, making it the third largest disease burden in the world after cardiovascular disease and cancer, causing economic losses to society and patients’ families (1, 2). The disability rate of stroke is as high as 60%–80% (2), among which post-stroke hemiplegia is the main reason for the high disease burden (3). Currently, there is no particularly effective clinical treatment. Post-stroke hemiplegia may result in permanent disability if they do not receive timely and effective treatment (3, 4). The high cost of traditional rehabilitation, coupled with the cumbersome rehabilitation training methods, family commuting, poor treatment effects, and other factors make it difficult for patients to adhere to treatment or the treatment effects are few (5). In recent years, innovative rehabilitation strategies to improve motor function, represented by deep brain stimulation, have emerged (6–9). Deep brain stimulation can provide long-term chronic stimulation, and adjust the stimulation parameters during follow-up (10–12). Fine-tuning the treatment has achieved better therapeutic effects in many diseases that were difficult to treat in the past, such as Parkinson’s disease and depression (13, 14). Therefore, it has been used to improve motor function recovery after stroke and has been confirmed by human clinical studies. In a phase I clinical trial conducted by Baker et al. in 2023, 12 patients with moderate to severe upper limb motor deficits after unilateral stroke received cerebellar dentate nucleus-DBS treatment (7, 15). After treatment, the patient’s upper limb Fugl-Meyuer motor function scores improved by 7 points, the motor function of the upper limbs was significantly improved, and no serious complications occurred during the treatment (7).

**Figure 1:**
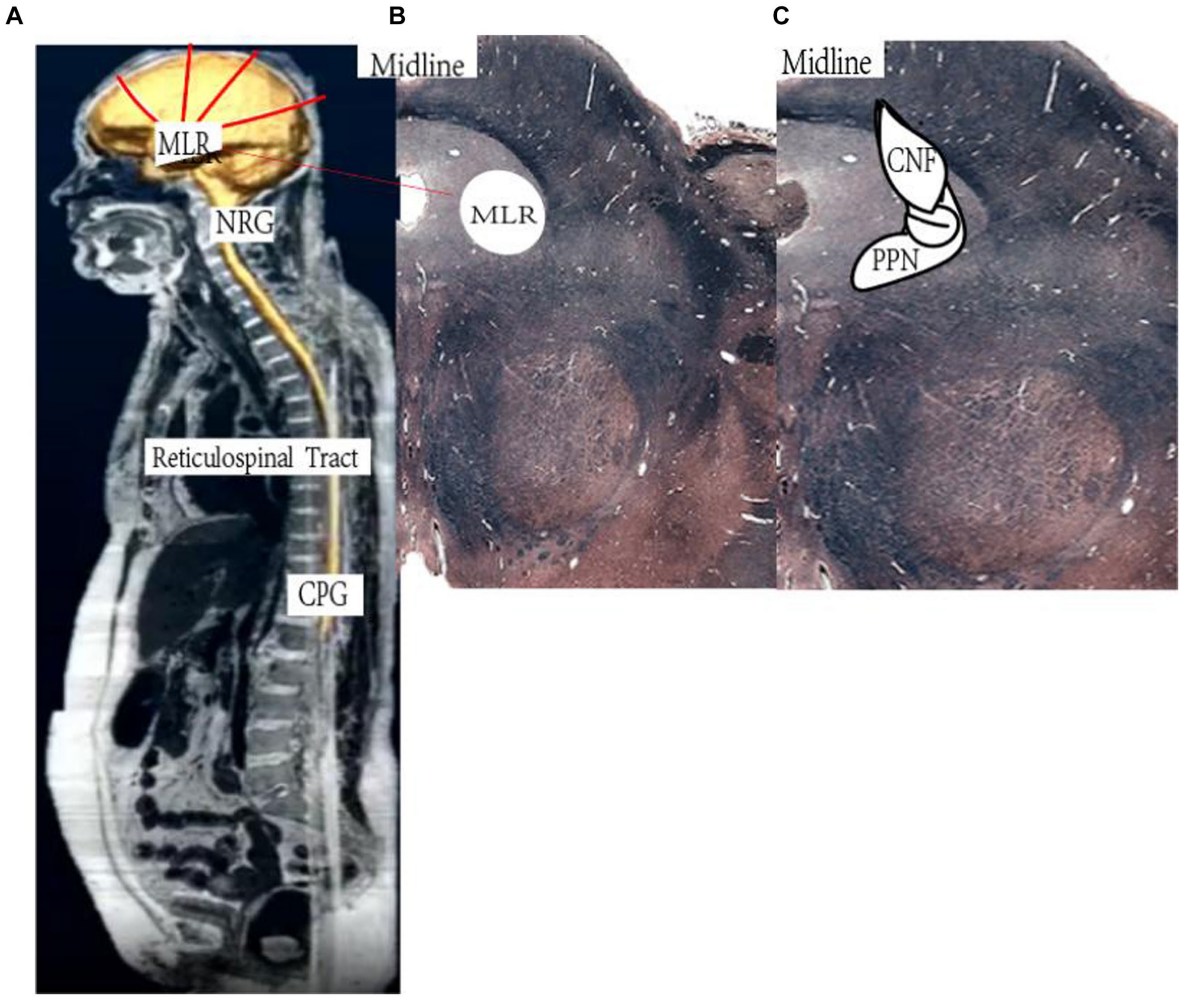
Schematic illustration of the reticulospinal system. **(A)** Higher central nervous system centers of motion control send their signals to the mesencephalic locomotor region (MLR). The MLR is bilaterally linked to its downstream target, the gigantocellular reticular nucleus (NRG), which gives rise to the reticulospinal tract and drives the central pattern generators (CPG) for motoneuron activation and locomotion. **(B,C)** Horizontal section of the human **(B)** and cross section of the rat **(C)** midbrain at the level of the superior colliculi depicting the MLR (**B**: landmarks based on Afshar et al. 90; **C**: landmarks based on Paxinos and Watson 91). CNF, cuneiform nucleus; PPN, pedunculopontine nucleus. Based on https://www.neuroanatomy.ca/index.html.

The mesencephalic locomotor region (MLR) is critical for motor recovery (16–19). The MLR (Figure 1) is a phylogenetically conserved key motor control center in the brainstem, which is composed of two leading nuclei, namely the Pedunculopontine Nucleus (PPN) and the cuneate nucleus (CNF) (20–23). PPN is associated with exploratory behavior, and deep brain stimulation (DBS) of PPN in patients with Parkinson’s disease can reverse the freezing of gait (16, 23–28). CNF is the main control area for movement initiation, maintenance, and speed regulation, and research on stimulating this target with DBS to improve incomplete spinal cord injury (SCI) has aroused scientific and clinical interest (29, 30). Electrical stimulation of the rat MLR in an acute rodent stroke model to achieve near-physiological hindlimb movements during walking and swimming (20), and MLR-DBS improved walking speed and limb coordination (31). The MLR-DBS study confirmed that MLR-HFS does not affect the infarct area, however, it can regulate the area around the lesion and reduce neuroinflammation by affecting the cholinergic anti-inflammatory pathway (32–35). In particular, CNF high-frequency stimulation (HFS) can significantly reduce the neuroinflammation at the stimulation site. Inflammatory cytokines and chemokines, reduce the concentration of pro-inflammatory cells (23, 33). In addition, DBS can stimulate CnF glutamatergic neurons, activate their afferent or efferent fibers, and stimulate spinal reticular fibers to initiate movement within a short latency period (32, 33, 36–39). Judging from the above literature, MLR-DBS is a potentially effective treatment for post-stroke motor dysfunction, but the application of DBS in the treatment of post-stroke sequelae is still in the exploratory stage, and a large number of studies are still in the animal experiment and clinical verification stages (40). No large-sample clinical trials have been conducted. So we designed this multi-center randomized controlled clinical trial (RCT) (7). The leading purpose is to explore the safety and effectiveness of DBS in improving motor function in patients with post-stroke hemiplegia. The secondary purpose is to explore its potential therapeutic mechanism and provide clinical evidence support for the widespread application of DBS technology in post-stroke hemiplegia.

## Methods and analysis

### Study design

**Figure 2:**
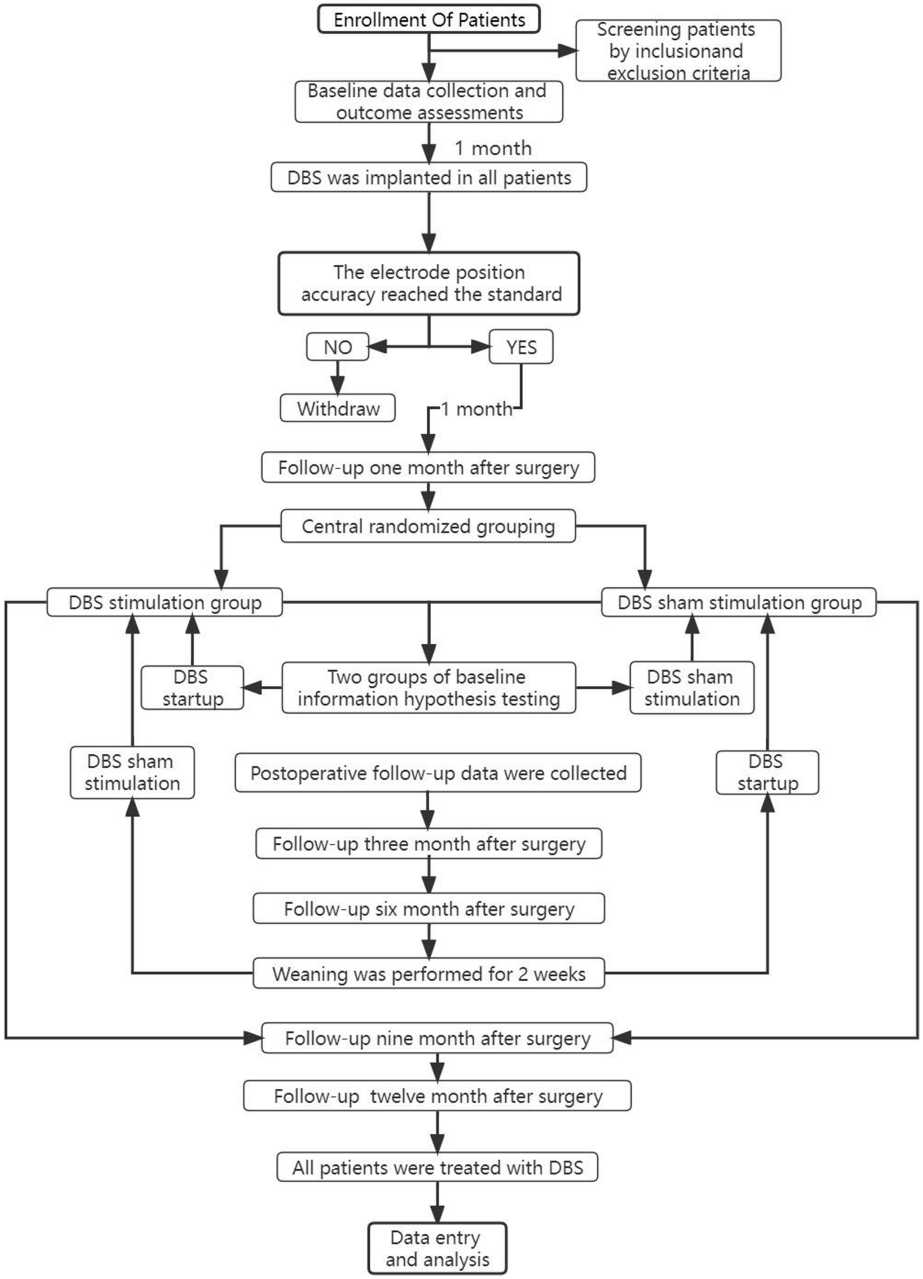
Brief flowchart of the entire study with draw. For patients whose electrode is not implanted in the ideal position in CT review after DBS implantation, the device can be temporarily left off, and the follow-up of clinical trials is excluded, and relevant conventional rehabilitation means can be actively given. The health status of patients can be observed at any time, and regular follow-up can be conducted. When all clinical trials are over, the effect of DBS treatment can be observed. If the effect of DBS treatment is better than that of the traditional control group, the electrode position should be adjusted again and DBS treatment should be performed again.

The trial is a multicenter, prospective, double-blind crossover randomized controlled trial (RCT). The overall process is in Figure 2. In this study, we will include 62 moderate to severe post-stroke hemiplegia patients who had stable disease after 1 year of conservative treatment in multiple neuro medical centers in the General Hospital of the People’s Liberation Army of China et al. All patients included in the study will undergo deep brain electrode implantation. They will be randomly postoperatively divided into a DBS group and a control group, with 31 patients in each group. 1 month after surgery, the DBS group will be given electrical stimulation treatment, while the control group will undergo routine observation. Following up in the 3rd and 6th months after surgery. After the follow-up, all patients will stop electrical stimulation treatment for 2 weeks. After the 2-week washout period, the control group will start electrical stimulation treatment, and the DBS group will continue observation. Outpatient follow-up in the 6th and 9th months after surgery. After the follow-up, all patients will undergo electrical stimulation treatment. Observe for a long time. The leading outcome measure of follow-up is the pre-and post-change score on the Fugl-Meyer Assessment Upper Extremity Scale (FMA-UE). In addition, we will follow up on changes in the secondary outcomes of balance function, neuropsychiatric behavior, Post-stroke Spasticity, fall risk, activities of daily living, and quality of life.

### Methods

#### Sample size

This study is a randomized, double-blind, active sham stimulation control design, and statistical experts determined that the sample ratio between the DBS group and the control group was 1:1. A review of the literature revealed that subjects’ upper limb FMA scores improved by an average of 7 points. Since this study was based on the changes in the mean FMA before and after DBS treatment, based on literature data, the mean FMA before DBS treatment was 21.92 ± 6.30, and the mean FMA after DBS treatment was 35.42 ± 12.23. This study is designed as a difference test to determine whether the mean of the DBS group (u1) is different from the mean of the control group (u2) (41, 42). The assumptions are as follows: H0: u1 − u2 = 0, H1: u1 − u2 ≠ 0. The two-sided significance level was 5%, the statistical power was 90%, and the dropout rate was 20% (43). The total sample size required for this study was calculated by PASS V.15 sample size calculation software to be 48 patients, with 24 people in each group. According to the possibility of electrode implantation deviation and other special circumstances leading to a lose, we included 62 patients, 31 in each group.

#### Enrollment

Researchers will publish recruitment information through the hospital website, designate dedicated personnel to consult patients and their families via telephone and video connections, conduct preliminary screening based on the exclusion criteria (Table 1), and recommend qualified patients to bring complete relevant examinations, test data, and cases to the hospital. In the outpatient clinic, qualified neurosurgery clinicians will further evaluate and diagnose the patients. Eligible patients will be enrolled, and the researcher will give full informed consent and sign an informed consent form. The evaluators will collect surgical baseline data based on the case report form (CRF). The patient will be admitted to the hospital 1 week before the operation. After admission, general preoperative examinations and tests will be completed, including an electrocardiogram, chest X-ray, hematuria and stool routine, coagulation screening, blood type identification, biochemistry, and eight preoperative items. Then, the patient will be evaluated by professional evaluators. The main evaluation contents will include an assessment of motor function, mental behavior, quality of life, past disease history, and related treatment drugs and measures. Finally, the day before the operation, the examiner will arrange for the patient to undergo a brain MRI examination. The examination results will be burned into a CD and handed over to a dedicated person for safekeeping. On the day of the operation, the surgical planning system (ELEKTA) will be introduced for preoperative planning, including electrode implantation paths and treatments (44).

**Table 1:** List of inclusion and exclusion criteria.

Inclusion	Exclusion
(1) Meet WHO or International diagnostic criteria for post-stroke hemiplegia;	(1) Glasgow Coma Scale (GSC) score below 15, Minimum Mental State Examination (MMSE) assessment for dementia indicated, suffering from mental disturbance and unable to cooperate with examination or treatment.
(2) The first unilateral supratentorial ischemic stroke, the condition is stable after acute treatment of ischemic stroke, the course of disease is ≥1 year, and participate in 2 evaluations (screening and baseline) before enrollment.	(2) Motor and sensory disturbances are not induced by stroke, nor by previous ischemic stroke, but stroke induced by trauma, brain tumor, etc.
(3) Diagnosed by professional physicians combined with brain CT or magnetic resonance imaging and other imaging techniques;	(3) Serious comorbidities, such as malignant tumors, primary heart, liver, kidney or hematopoietic system diseases.
(4) Between the ages of 18 and 80, male or female	(4) History of cognitive impairment, mental disorder, drug abuse, drug allergy, and alcoholism.
(5) The responsible lesion in the unilateral white matter area indicated by cranial CT or MRI	(5) Previous craniotomy, thrombectomy and thrombolysis.
(6) Relevant sequelae such as limb dysfunction after stroke, accompanied by unilateral limb motor dysfunction, proved to be right-handed by standardized examination.	(6) Possess a pacemaker, metal stent, plate, or implant susceptible to electrical impulses in the body (pacemaker or defibrillator, baclofen pump, deep brain stimulator, Ventricular shunts, shrapnel, etc.).
(7) There is obvious motor disorder, FMA motor function score is between 50 and 84;	(7) Pregnant or breast-feeding or have a recent birth plan.
(8) Perfect clinical data	(8) Clinical data are perfect.
(9) Stable medical and physical condition with adequate nursing support and appropriate medical care in the patient’s home community.	(9) Congenital or acquired abnormalities of lower extremities (affecting joints and bones).
(10) The patient himself or voluntarily signs the informed consent and is willing to cooperate with relevant treatment.	(10) Registration of investigators, their family members, employees, and other dependents.
Withdrawal criteria	(11) Severe joint contractures cause loss or limitation of lower limb activities.
(1) After the start of the clinical study, it is found that the subjects do not meet the case inclusion criteria;	(12) Blood system diseases with increased risk of bleeding during surgical intervention.
(2) There is no data after enrollment;	(13) Participate in another study drug study within 30 days before and during this study.
(3) Subjects have poor compliance and have never used the experimental treatment plan;	(14) Unable to complete the basic process, or difficult to maintain compliance and follow-up.
(4) Those who seriously violate the experimental protocol.	
Termination criteria	
Continuation of the study may harm the relevant rights and interests of a certain number of subjects	

#### Interventions

**Figure 3:**
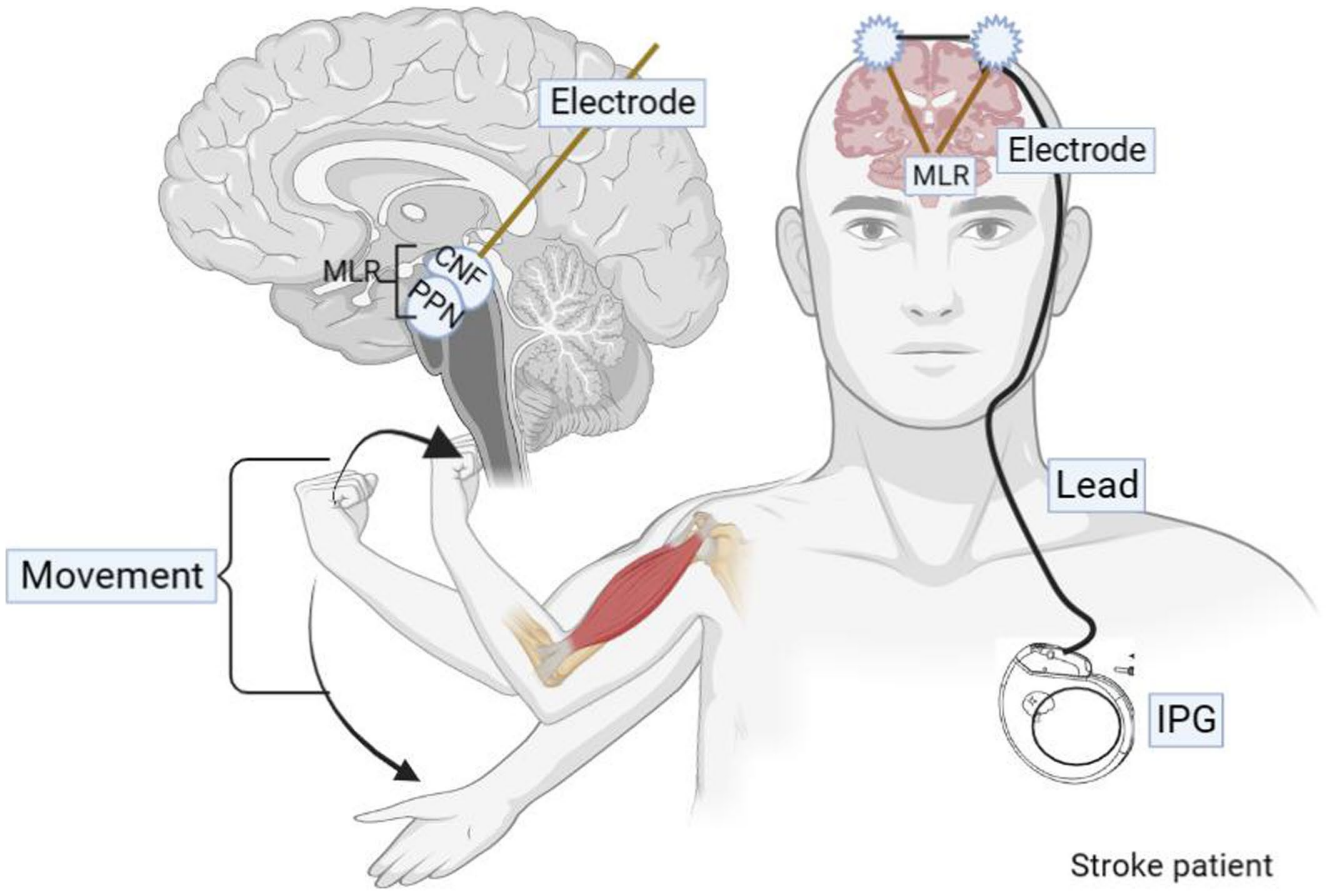
Graphic of DBS delivery during therapy sessions. During the in-clinic rehabilitation session, for each movement effort, the clinician/therapist generates the DBS pulse via a push button connected to a laptop computer. A Wireless Transmitter, attached to the laptop, triggers the patient’s implanted pulse generator to deliver the DBS pulse.

**Figure 4:**
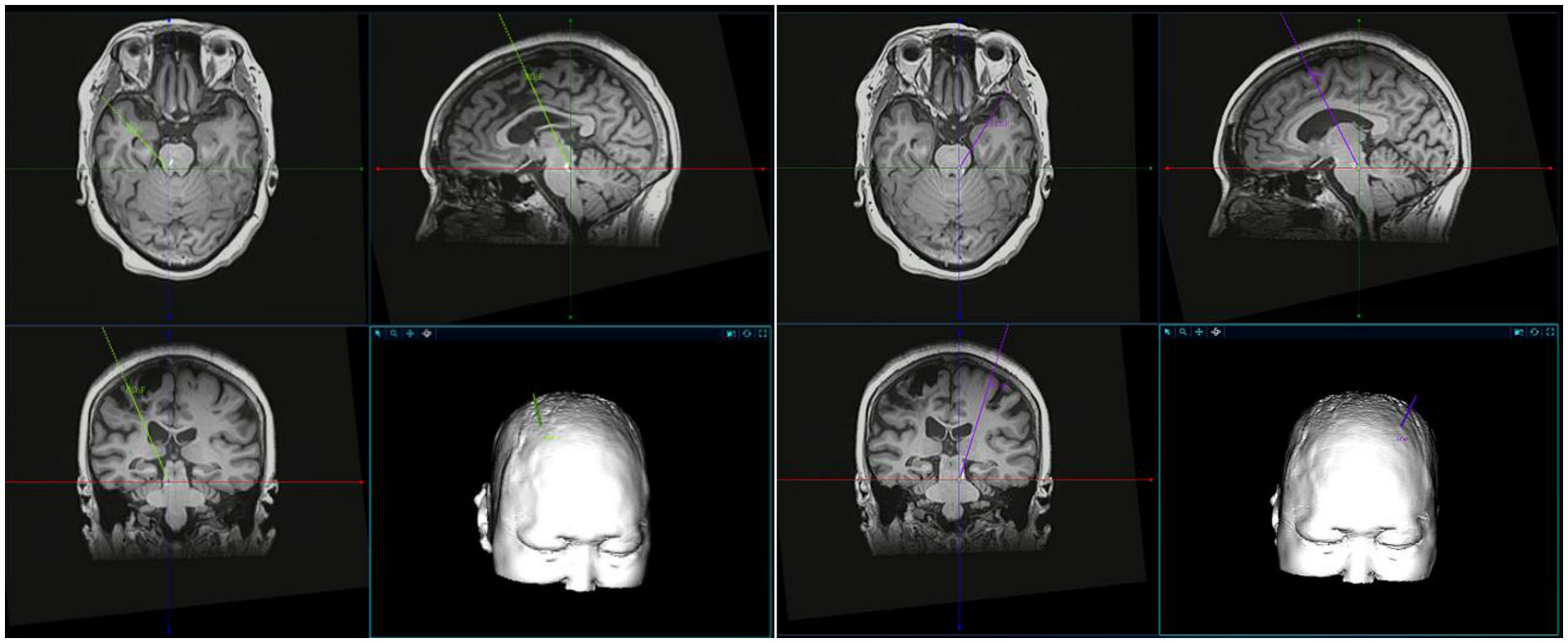
A schematic representation of the MLR localization. MLR localization was performed in combination with preoperative imaging, stereotactic map of human brain and related literature.

The process of MLR-DBS for post-stroke hemiplegia is in Figure 3. All individuals in the study will receive bilateral stereotactic implantation of intracranial leads under general anesthesia. The patient will be installed with a Leksecl-G directional device under general anesthesia in the operating room and undergo MRI positioning scanning in the MRI room during the operation. Default CnF coordinates will be calculated from the MRI brainstem landmarks to target the brainstem normalized coordinates and use the diffusion tract map to ensure we target the medial, superior cerebellar, and central peduncles, within the area demarcated by the lid (Figure 4). Before DBS lead placement, microelectrode recording and test stimulation will be performed to assess intraoperative physiology and rule out potential side effects that may require repositioning of the lead. First, electrophysiological mapping of CNF will be performed. Microelectrodes will be precisely inserted along predefined trajectories, directed toward the CNF, and connect the neuromodulation system and manual actuators to the stereotaxic device. The center of this area shows neuronal responses to imaginary walking, which stimulates passive and active lower body movements while the patient performs a series of motor tasks with their lower body suspended from the operating table. As this study is the first to investigate DBS of CNF in patients with post-stroke movement disorders, there are no guidelines for optimal stimulation parameters. However, comparable evidence accumulated from preclinical studies in various animal models suggests that low-frequency stimulation (≤50 Hz) at moderately wide pulse widths (200–1,000 μs) may be possible due to the evolutionary conservation of the MLR in mammalian species properties (45). Therefore, we first stimulated with increasing voltage at 20 Hz, 400 μs, 2.0–4.5 V pulse width, and then adjusted the frequency and pulse width based on individual intraoperative behavioral responses. Intraoperative MRI and postoperative CT will be used to determine whether the electrode insertion position will be deviated and whether the operation will be completed after checking for accuracy.

#### Randomization

The 62 patients will be enrolled in the group undergoing deep brain electrode implantation. The 62 patients will be randomly divided into two groups at a ratio of 1:1 using a simple random grouping method, with 31 patients in the DBS group and the control group. First, random numbers will be automatically generated by the OpenClinica clinical trial data management platform to achieve centralized randomization. When a new patient meets the inclusion and exclusion criteria and signs the informed consent form, each research center can apply for the patient’s randomization number after filling in the patient’s basic information in OpenClinica. The random numbers will be automatically issued by the system, and only one coordinator in each center has access. All patients included in the study will receive DBS implantation after baseline assessment and were then randomly divided into two groups in a 1:1 ratio: DBS treatment group and control group. The former will start electrical stimulation treatment 1 month after the operation. Specialists will assess the patient’s recovery status through the remote rehabilitation system every week and guide rehabilitation training and electrical stimulation treatment. The latter will receive the same rehabilitation training under the face-to-face guidance of experts, except for the power-on (sham stimulation, the power-on stimulation parameter was 0). Experts will evaluate the patient’s recovery and adjust rehabilitation strategies every week in the outpatient clinic. The study is blinded to participants, scale raters, and data analysts. Allocation information will be kept in opaque, sealed envelopes by a designated person not involved in the study, thus ensuring the concealment of random assignment.

#### Blind method

To ensure study quality, all operators, therapists, patients, evaluators, and analysts will remain blinded to assignments and interventions until data collection, analysis, and video recording are completed. During video recording, patients wear surgical caps to conceal the data collection time point. These videos will be uploaded to a central unit and scored by two neurologists, without knowledge of whether the patient is receiving DBS treatment. The data administrator will label the groups as A and B for analysis. However, to safeguard patient safety, the operating doctor cannot be blinded to provide timely treatment in case of emergency. Overall, patient safety is our utmost priority (44).

#### Outcome measurements

All outcome measures will be evaluated by professional research assistants 1 week before surgery, during the perioperative period, and at one-month, three-month, six-month, nine-month, and 12-month intervals postoperatively (Table 2). All measurement results will be securely uploaded to a third-party online data management system. The primary outcome measure is the improvement in patients’ motor function before and after deep brain stimulation, assessed primarily through changes in the Fugl-Meyer Assessment Upper Extremity Scale (FMA-UE). Secondary indicators include the evaluation of patients’ balance function, neuropsychiatric behavior, fall risk, activities of daily living (ADL), and quality of life before, after, and at various time points following treatment, along with the calculation of the stroke improvement rate.

**Table 2:** Participant timeline of data collection.

Study period	Screening	Perioperative period		Follow-up period				
	Enrollment	Allocation/surgery	Postsurgery	1 month	3 month	6 month	9 month	12 month
Number of days	15 ± 7	10 ± 7	3 ± 2	7 ± 3	7 ± 3	7 ± 3	7 ± 3	7 ± 3
**Enrollment**								
Eligibility screen	×							
Informed consent	×							
Medical history	×							
MRI scanning	×		×					
Interventions								
MLR-DBS surgery		×						
Stimulation parameters		×						
**Allocation**								
DBS group				×	×	×		
Control group							×	×
**Assessments**								
**Primary outcome measure**								
FMA	×		×	×	×	×	×	×
**Secondary outcome measures**								
BBS	×		×	×	×	×	×	×
POMA	×		×	×	×	×	×	×
BI	×		×	×	×	×	×	×
SF-36	×		×	×	×	×	×	×
HAMD	×		×	×	×	×	×	×
AE		×	×	×	×	×	×	×

### Main outcome

#### Motor function intervention effect

The main evaluation index of this study is the improvement of patients’ motor function before and after deep brain stimulation, which is being evaluated through changes in the Fugl-Meyer Assessment Upper Extremity Scale (FMA-UE) (7). FMA-UE is widely used in clinical motor function assessment and is a quantitative stroke-specific scale used to assess motor function, balance, sensation, and joint function in patients with hemiplegia. Each of the five areas contains different assessment items that are scored on a 3-point scale. This scale has good validity and reliability in the stroke population. The scale covers 50 items, with a total score of 226 points (46). There are 17 sports items, with a total score of 100 points, of which the upper limb motor function score is 66 points, the lower limb motor function score is 34 points. The higher the assessment score, the better the patient’s motor function (47).

### Secondary outcome

#### Balance ability

Stroke patients suffer from balance dysfunction due to impaired control of the brain’s central nervous system and sensory or motor conduction pathways. The Berg Balance Scale (BBS) is mainly used to evaluate the balance and coordination abilities of participants during intervention and follow-up (48, 49). This scale is suitable for patients with strokes of various severity. Through assessment, it can identify whether patients need help with their movements and prompt the patient’s prognosis. It is mainly used in subacute and chronic strokes to predict and evaluate balance after stroke. It has reliability and validity, sensitivity is good, and it is currently the most commonly used international balance scale for stroke patients. The form includes a total of 14 items, each item includes 5 levels (0 to 4 points), with a total score of 56 points. Higher scores indicate better balance ability (49, 50).

Post-stroke Spasticity will be evaluated using the modified Ashworth Scale (MAS), a widely-used assessment method. It measures resistance during passive joint movements, especially suitable for patients with hemiplegia. Muscle spasticity is graded from 0 to 4, with higher grades indicating increased tone. MAS has proven reliable and valuable in clinical settings for assessing upper limb muscle spasticity. Notably, up to 97% of chronic stroke patients with moderate to severe motor impairments experience spasticity (51–53).

#### Fall risk

Falls after stroke are common sequelae of poor motor function recovery, which can cause fear of walking, lead to prolonged hospitalization, increase the risk of disability and death, and seriously affect the independence and quality of life after stroke. Performance-oriented activity assessment (POMA) will be used to assess fall risk during intervention and follow-up. A previous study reported that the POMA is an easy-to-administer task-oriented test with higher test weight than other tests such as the time remaining test, one-leg stance test, and functional accessibility test. Reliability, discriminant validity, and predictive validity (54, 55). The total possible score is 28, the lower the score, the greater the risk of falling (56).

#### Activities of daily living

About 75% of stroke patients suffer from varying degrees of disability, and in severe cases, they even lose the ability to take care of themselves (57). In the process of stroke rehabilitation, the training of self-care ability in daily life is also very important and necessary. Participants’ ability to perform activities of daily living will be assessed by the Barthel Index (BI) during the intervention and follow-up periods (58, 59). BI is a widely used clinical assessment method for daily living ability. It can be used not only to evaluate functional status before and after treatment, but also to predict treatment effects, length of stay, and prognosis. It has the advantages of simple evaluation, strong operability, high reliability, and high sensitivity. Previous studies have shown that BI has high internal and external reliability in assessing the daily living activities of stroke survivors. BI consists of 10 items describing different activities, with a total score of 100 points. The higher the score, the better the patient’s independence and the lower the dependence on others.

#### Quality of life

Patients with improved motor function are often accompanied by improved quality of life. Effective rehabilitation treatment can improve the quality of life after stroke, help patients restore function, improve self-confidence and self-esteem, and reintegrate into social life. This study mainly used a 36-item short-form questionnaire (SF-36) to assess patients’ quality of life. The scale includes 8 aspects and 36 items (60–62). It is easy to use, accepted by patients and meets strict reliability and validity standards. A higher assessment score represents a higher quality of life for the patient.

#### Psychological improvement

Patients with post-stroke hemiplegia may develop post-stroke depression (PSD), which is characterized by persistent depressed mood disorder (mood disorder). The overall incidence rate is as high as 40% to 50%, of which about 15% are severe depression, which may be accompanied by severe suicide or even suicidal behavior. This study used the Hamilton Depression Rating Scale (HAMD) for evaluation (63). This scale is simple to operate, has high reliability and validity, and is widely used in clinical applications. Among them, the HAMD-24 is the most clinically used. Therefore, this study evaluated patients before and after HAMD-24 treatment. A total score of more than 8 points indicates the presence of depressive symptoms in patients, and the higher the score, the more severe the depressive symptoms.

#### Adverse events

During the treatment process, a small number of patients may experience symptoms such as accidental injuries, skin rupture and bleeding, muscle soreness, joint pain, and rashes. Any adverse events (AEs) during recovery should be documented in a specific CRF with time of occurrence, severity, relationship to the duration of the intervention, and prognosis. Investigators should take necessary measures to deal with all AEs to ensure the safety of the subjects and follow up with the subjects until their physical condition returns to normal levels. All adverse events (such as pain and falls) occurring during intervention and follow-up will be recorded on a case record form (CRF) through monitoring and self-reporting, and the relevance of the intervention will be assessed. The incidence of an adverse event was defined as the number of patients in whom it occurred divided by the number of all patients. Calculate the number and frequency of adverse events, related adverse events (adverse reactions), adverse events leading to withdrawal, and serious adverse events, and provide detailed records (64, 65).

## Data management and analysis

### Data management

Before the study begins, all researchers receive comprehensive training on the protocol so that subjects have a clear understanding of the testing requirements subjects can better cooperate and provide more realistic information during the testing process. During the follow-up period, oral medications can be needed, but medications that may significantly interfere with the study results are prohibited from being used as auxiliary treatment. All medications should stopped 24 h before each follow-up assessment. If discomfort during the period, a remote program control should be carried out in time to adjust the stimulation parameters. The follow-up method is mainly outpatient follow-up. When irresistible factors occur, telephone contact and WeChat video connection can be temporarily used instead. However, during subsequent outpatient visits, all information obtained from the last non-outpatient visit must be shared with the patient. Check with your family members to ensure the authenticity and accuracy of the information. All researchers will use unified, standardized, normative, and internationally accepted assessment scales and case report forms (CRF). Before surgery, perioperatively, and 1 month, 3 months, 6 months, 9 months, and 12 months after surgery, blinded professional evaluators strictly followed the standardized evaluation scale and evaluation process to conduct evaluations and record standardized videos. Professional clinicians not related to this study will re-evaluate the scores at each follow-up time point and compare the scores obtained with the first score. If the difference is not significant, the first evaluation results will used. If the difference is too big, a third evaluation will performed. Once the assessment results are accurate, the recorder uploads the data to the case report form. During the data collection process, two data supervisors will review the contents of CRF to ensure the authenticity and accuracy of the data. If there are data errors or incomplete information, the data manager sends it back to the appropriate center, where the researcher checks the original file, rechecks, and updates the data. At the end of the trial, the data administrator will lock the database and send the data separately to two data analysts for analysis. During the research process, to ensure the quality and accuracy of the data, only designated personnel not related to this study can view and modify the data, and each modification will be recorded, and personnel related to this trial will remain evasive throughout the process.

### Data analysis

All data analysis will based on IBM SPSS Statistics 25.0, describing the total score of each scale at each follow-up and the change from baseline at each visit after treatment. Measurement data using mean ± standard deviation to describe. Count data Use numerical values (%) to describe, and use group t-test or Wilcoxon to compare between groups according to the characteristics of the data. Intra-group comparisons were performed using rank sum-test, paired t-test or paired signed rank-test. For comparisons of pre-and post-treatment change values based on baseline general information, appropriate multivariate analysis models were used to correct for the effects of confounding factors or covariates based on the data situation. The single-item count data of each scale used the chi-square test or the exact probability test, and the grade data used the Wilcoxon rank sum test. A paired rank sum test and repeated measures analysis of variance were used to analyze the changes in score data within the group before and after treatment. The effectiveness measurement index is the improvement rate of each clinical scale at different follow-up times = (scale score at each postoperative time point − preoperative score) / preoperative score * 100%. A score improvement rate > 25% is considered effective. Safety observation indicators are all postoperative adverse reactions, including surgery-related adverse reactions, device-related adverse reactions, and stimulation-related adverse reactions. The type, number of cases, occurrence time, treatment measures, and prognosis of adverse events are recorded. The incidence of adverse events is defined as the number of patients in which it occurs divided by the number of all patients, and an adverse event rate of less than 5% is considered safe.

### Data monitoring

The entire implementation of this study will be regularly supervised and guided by specialized personnel assigned by the Ethics Committee of the People’s Liberation Army General Hospital who have no interest in this study. All information related to adverse (AEs), protocol revisions, and protocol deviations will be reported to the Ethics Committee. During enrollment and follow-up evaluation, a third-party data management company assigns dedicated personnel to supervise to ensure the accuracy and objectivity of the evaluation. After the evaluation, a third-party data management company assigned a dedicated person to conduct data verification. After verification, two blind research assistants entered all the collected data into the online data management system through double-entry. Before conducting descriptive and statistical analyses, data checks (CRF checks, and duplicate checks on raw data) were performed to ensure data accuracy. Access to the data set is restricted to clinical trial management and the data safety and monitoring board. The storage and processing of research data will strictly comply with the regulations and policies of the researcher’s institution and research site. In addition, serious adverse events will be reported in detail to the Ethics Committee of the PLA General Hospital and the independent Data Safety and Monitoring Committee, which will recommend whether to continue, modify, or stop the intervention. During the research process, data sets will be stored, analyzed, and archived pseudonymously to protect personal privacy.

### Research difficulties

A particular challenge remains trajectory planning and lead implantation. Compared with the rodent PPN and CNF, many regions of the human brainstem, including MLR subnuclei, are small and poorly described. Based on known coordinates in PPN DBS, the frozen gait symptom in Parkinson’s disease patients can be successfully reduced to landmarks in human and rodent stereotaxic atlases to map the relationship of CNF to PPN. To improve the accuracy of planning trajectories and intraoperative positioning, a more detailed description of the macroscopic and microscopic anatomy of human MLR is urgently needed.

## Discussion

The study is currently being conducted at multiple centers including the Chinese People’s Liberation Army General Hospital. At present, research on the application of DBS in post-stroke motor dysfunction is gradually carried out (66). Slotty et al. (67) used GPi-DBS combined with Vim/ventral nucleus (Vop)-DBS to treat a case of unilateralism secondary to putamen stroke. In studies on patients with dystonia, the results showed that the patient’s motor symptoms were significantly and sustainably improved; Elias et al. included nine studies with a total of 32 patients with post-stroke dyskinesia and found that at least 13 of the patients received DBS treatment (12). Post-motor symptoms improved significantly. Koerbel et al. (68) reported a 48-year-old patient after a thalamic hemorrhagic stroke. After DBS zona incerta (ZI) treatment, the patient’s proximal motor function was significantly improved, the abnormal involuntary movement scale score was significantly reduced. Franzini et al. (15) reported that the motor function of 3 patients with post-stroke motor function deficits improved after DBS treatment of the contralateral internal capsule posterior limb. Baker et al. (7) used cerebellar dentate nucleus-DBS to treat 12 patients with moderate to severe upper limb motor deficits after unilateral stroke. The patients’ upper limb Fugl-Meyuer motor function score increased by 7 points, and the upper limb motor function improved. There was no improvement during the treatment. Serious complications occur. For patients with post-stroke motor dysfunction, the range of stimulation parameters is wide and needs to be adjusted individually according to the stimulation target. Existing literature shows that the optimal stimulation parameters must be determined individually for each patient. The optimal stimulation parameters have not yet been established. The conclusion is still under intense discussion. Paro et al. (12) systematically analyzed 82 DBS electrodes implanted in 53 patients and found that the voltage [M (range)] of 55 leads was 3.4 (2.4~4.0) V, the frequency [M (range)] of 63 leads is 145 (130~185) Hz, and the pulse width [M (range)] is 90 (60~120) μs. However, there are also studies showing that wider pulses (>400 μs) appear to be more effective in enhancing movement and more convenient than shorter pulse widths, and preliminary results from local field potential (LFP) measurements and behavioral tests suggest that lower stimulation frequencies (8–20 Hz) are more appropriate, therefore, we first stimulated with 20 Hz, 400 μs, 2.0–4.5 V pulse width, increasing voltage, and then adjusted the frequency and pulse width according to individual intraoperative behavioral responses. Although a large number of relevant studies have been carried out to initially confirm the unique advantages, effectiveness, and safety of DBS in patients with post-stroke hemiplegia, they are still at the stage of basic animal experiments, case reports and case series reports and large-sample RCTs have not yet been carried out. Research to confirm its effectiveness and safety. Based on this, we designed this research protocol. To our knowledge, this trial protocol is the first to explore the effect of MLR-DBS on improving motor function in stroke survivors. It is also the first RCT in the field of application of DBS in post-stroke motor dysfunction. Research proposal. This multicenter, prospective, randomized, double-blind crossover controlled clinical research protocol is the first to develop and execute a study on the safety, feasibility, and safety of deep brain stimulation (DBS) in the midbrain motor region (MLR) to improve motor dysfunction after stroke plan. The evaluation results were evaluated by random shuffling, and the first evaluation was blindly evaluated with standardized videos for comparison before and after to reduce potential bias. The randomization process of this study was carried out after the surgery of all patients, thus avoiding to a large extent the interference on the study results caused by the surgeon’s inability to implement blinding. Our research team has performed thousands of deep brain electrode implantation surgeries to treat Parkinson’s disease, dystonia, Major syndrome, and other diseases, and has rich clinical and surgical experience. The main purpose of this trial protocol is to improve wheelchair, subacute, and chronic stroke motor dysfunction through MLR-DBS and to study the clinical feasibility and efficacy of MLR-DBS in humans. Study results will demonstrate the effectiveness, efficacy, and safety of MLR-DBS in improving motor function in stroke survivors. Our goal is to maximize the long-term restoration of lost motor function in patients with severe movement disorders. This research program will guide on the importance of integrating existing and different rehabilitation techniques and designing more effective rehabilitation programs. The results of this trial will help to understand the neural mechanisms of MLR-DBS in stroke patients who develop hemiparesis after stroke. Provide clinical evidence support for the large-scale clinical application of DBS technology.

At the same time, this research protocol also has some shortcomings and the following areas that need improvement. First, the sample size is small and limited to the chronic phase of stroke patients, so the results cannot be generalized to the wider stroke population. Spontaneous recovery and potential complications of stroke heterogeneity are more evident in patients with acute and subacute ischemic stroke, and further studies in a larger number of patients may be warranted. Secondly, the sham operation group in our study did not receive electrical stimulation. For the sake of patient interests and ethical requirements, the sham stimulation group will not last for a long time and cannot completely imitate real-world research. This configuration is a sham. The weakest version of the control group, since electrical stimulation is likely to be detected in the active DBS group, while in the sham group, there is no electrical stimulation. Third, open-loop DBS was applied in our study, but closed-loop neuromodulation is more effective than open-loop neuromodulation, and closed-loop DBS should be investigated in future studies. Fourth, the ideal stimulation parameters of DBS are one of the most critical challenges for its application, as these parameters have a huge impact on clinical efficacy. The optimal timing of DBS initiation and many optimal parameters such as stimulation site and side, electrode and waveform configuration, efferent or afferent stimulation, and titration regimen are unknown. Our treatment parameters may affect treatment outcomes. In the future, larger-scale clinical trials and clinical practice applications are needed to confirm the actual application effect of DBS. It is necessary to discuss treatment parameters in future studies.

## Advantages and limitations

### Advantages

This multicenter, prospective, randomized, double-blind crossover clinical study is the first to investigate the feasibility and safety of midbrain motor region (MLR) deep brain stimulation (DBS) in improving motor dysfunction after stroke. To minimize bias, we will use standardized videos for blinded evaluation and randomly disrupt the evaluation results before and after the initial assessment. The randomization process will occur post-surgery, ensuring surgeons’ blinding and preventing study interference. Our team has extensive experience in deep brain electrode implantation surgeries for Parkinson’s disease, dystonia, and other conditions. Additionally, we will conduct significant basic research on MLR-DBS, achieving promising results currently being compiled for publication.

### Limitations

While this surgery-related study has taken measures to minimize the surgeon’s impact on blinding, patient safety remains paramount. In cases of adverse reactions, urgent unblinding may be necessary, potentially affecting experimental accuracy.

### Dissemination

The findings will be published in a peer-reviewed journal, and the publication will be published on an “open access” clause, making the dataset accessible to research investigators and statistical evaluators. Research results will also be distributed to participants, stakeholders and policy makers (Beijing Municipal Commission of Economy and Informatization, Beijing Municipal Commission of Health and Family Planning). Researchers will be responsible for publishing the study, sharing the results with the wider scientific community, regardless of the size or direction of the effect.

## Ethics statement

The studies involving humans were approved by the Ethics Committee of the Chinese PLA General Hospital. All research procedures complied with the current version of the Declaration of Helsinki (see www.wma.net for details) and relevant ethical guidelines. The studies were conducted in accordance with the local legislation and institutional requirements. The participants provided their written informed consent to participate in this study. The research team will communicate study information, including study objectives, recruitment criteria, study protocol, potential risks, and expected functional benefits to recruiting participants. Participants will be informed that they may withdraw from the study at any time without consequences.

## Author contributions

JX: Investigation, Writing – original draft. BL: Investigation, Writing – review & editing. SL: Supervision, Writing – review & editing. ZF: Methodology, Software, Writing – review & editing. YZ: Formal analysis, Validation, Writing – review & editing. DL: Writing – review & editing. QC: Writing – review & editing. HY: Formal analysis, Writing – review & editing. YC: Conceptualization, Writing – review & editing. XY: Resources, Visualization, Writing – review & editing. ZM: Funding acquisition, Resources, Visualization, Writing – review & editing.

## References

[1] GBD 2019 Stroke Collaborators. Global, regional, and national burden of stroke and its risk factors, 1990-2019: a systematic analysis for the global burden of disease study 2019. Lancet Neurol. (2021) 20:795–820. doi: 10.1016/S1474-4422(21)00252-0, PMID: 34487721 PMC8443449

[2] Wang W Jiang B Sun H Ru X Sun D Wang L . Prevalence, incidence, and mortality of stroke in China: results from a Nationwide population-based survey of 480 687 adults. Circulation. (2017) 135:759–71. doi: 10.1161/CIRCULATIONAHA.116.025250, PMID: 28052979

[3] Feigin VL Owolabi MO. Pragmatic solutions to reduce the global burden of stroke: a world stroke organization-lancet neurology commission. Lancet Neurol. (2023) 22:1160–206. doi: 10.1016/S1474-4422(23)00277-6, PMID: 37827183 PMC10715732

[4] Mehanna R Jankovic J. Movement disorders in cerebrovascular disease. Lancet Neurol. (2013) 12:597–608. doi: 10.1016/S1474-4422(13)70057-7 23602779

[5] Winstein CJ Stein J Arena R Bates B Cherney LR Cramer SC . Guidelines for adult stroke rehabilitation and recovery: a guideline for healthcare professionals from the American Heart Association/American Stroke Association. Stroke. (2016) 47:e98–e169. doi: 10.1161/STR.0000000000000098, PMID: 27145936

[6] Bahr Hosseini M Hou J Bikson M Iacoboni M Gornbein J Saver JL. Central nervous system electrical stimulation for neuroprotection in acute cerebral ischemia: Meta-analysis of preclinical studies. Stroke. (2019) 50:2892–901. doi: 10.1161/STROKEAHA.119.025364, PMID: 31480966 PMC6756951

[7] Baker KB Plow EB Nagel S Rosenfeldt AB Gopalakrishnan R Clark C . Cerebellar deep brain stimulation for chronic post-stroke motor rehabilitation: a phase I trial. Nat Med. (2023) 29:2366–74. doi: 10.1038/s41591-023-02507-0, PMID: 37580534 PMC10504081

[8] Dawson J Liu CY Francisco GE Cramer SC Wolf SL Dixit A . Vagus nerve stimulation paired with rehabilitation for upper limb motor function after ischaemic stroke (VNS-REHAB): a randomised, blinded, pivotal, device trial. Lancet. (2021) 397:1545–53. doi: 10.1016/S0140-6736(21)00475-X, PMID: 33894832 PMC8862193

[9] Plow EB Machado A. Invasive neurostimulation in stroke rehabilitation. Neurotherapeutics. (2014) 11:572–82. doi: 10.1007/s13311-013-0245-y, PMID: 24353109 PMC4121447

[10] Elias G Namasivayam AA Lozano AM. Deep brain stimulation for stroke: current uses and future directions. Brain Stimul. (2018) 11:3–28. doi: 10.1016/j.brs.2017.10.005, PMID: 29089234

[11] Hermann DM Chopp M. Promoting brain remodelling and plasticity for stroke recovery: therapeutic promise and potential pitfalls of clinical translation. Lancet Neurol. (2012) 11:369–80. doi: 10.1016/S1474-4422(12)70039-X, PMID: 22441198 PMC3964179

[12] Paro MR Dyrda M Ramanan S Wadman G Burke SA Cipollone I . Deep brain stimulation for movement disorders after stroke: a systematic review of the literature. J Neurosurg. (2022) 28:1–14. doi: 10.3171/2022.8.JNS221334 36308482

[13] Chiken S Nambu A. Mechanism of deep brain stimulation: inhibition, excitation, or disruption. Neuroscientist. (2016) 22:313–22. doi: 10.1177/1073858415581986, PMID: 25888630 PMC4871171

[14] Wathen CA Frizon LA Maiti TK Baker KB Machado AG. Deep brain stimulation of the cerebellum for poststroke motor rehabilitation: from laboratory to clinical trial. Neurosurg Focus. (2018) 45:E13. doi: 10.3171/2018.5.FOCUS18164, PMID: 30064319

[15] Franzini A Cordella R Nazzi V Broggi G. Long-term chronic stimulation of internal capsule in poststroke pain and spasticity. Case report, long-term results and review of the literature. Stereotact Funct Neurosurg. (2008) 86:179–83. doi: 10.1159/000120431, PMID: 18334861

[16] Caggiano V Leiras R Goñi-Erro H Masini D Bellardita C Bouvier J . Midbrain circuits that set locomotor speed and gait selection. Nature. (2018) 553:455–60. doi: 10.1038/nature25448, PMID: 29342142 PMC5937258

[17] Capelli P Pivetta C Soledad Esposito M Arber S. Locomotor speed control circuits in the caudal brainstem. Nature. (2017) 551:373–7. doi: 10.1038/nature24064, PMID: 29059682

[18] Noga BR Whelan PJ. The mesencephalic locomotor region: beyond locomotor control. Front Neural Circuits. (2022) 16:884785. doi: 10.3389/fncir.2022.884785, PMID: 35615623 PMC9124768

[19] Ryczko D Dubuc R. The multifunctional mesencephalic locomotor region. Curr Pharm Des. (2013) 19:4448–70. doi: 10.2174/1381612811319240011, PMID: 23360276

[20] Bachmann LC Matis A Lindau NT Felder P Gullo M Schwab ME. Deep brain stimulation of the midbrain locomotor region improves paretic hindlimb function after spinal cord injury in rats. Sci Transl Med. (2013) 5:208ra146. doi: 10.1126/scitranslmed.3005972 24154600

[21] Josset N Roussel M Lemieux M Lafrance-Zoubga D Rastqar A Bretzner F. Distinct contributions of mesencephalic locomotor region nuclei to locomotor control in the freely behaving mouse. Curr Biol. (2018) 28:884–901.e3. doi: 10.1016/j.cub.2018.02.007 29526593

[22] Joutsa J Horn A Hsu J Fox MD. Localizing parkinsonism based on focal brain lesions. Brain. (2018) 141:2445–56. doi: 10.1093/brain/awy161, PMID: 29982424 PMC6061866

[23] Zörner B Bachmann LC Filli L Kapitza S Gullo M Bolliger M . Chasing central nervous system plasticity: the brainstem's contribution to locomotor recovery in rats with spinal cord injury. Brain. (2014) 137:1716–32. doi: 10.1093/brain/awu078, PMID: 24736305

[24] Goetz L Bhattacharjee M Ferraye MU Fraix V Maineri C Nosko D . Deep brain stimulation of the Pedunculopontine nucleus area in Parkinson disease: MRI-based Anatomoclinical correlations and optimal target. Neurosurgery. (2019) 84:506–18. doi: 10.1093/neuros/nyy151, PMID: 29846707

[25] Moro E Hamani C Poon YY al-Khairallah T Dostrovsky JO Hutchison WD . Unilateral pedunculopontine stimulation improves falls in Parkinson's disease. Brain. (2010) 133:215–24. doi: 10.1093/brain/awp261, PMID: 19846583

[26] Pereira EA Muthusamy KA De Pennington N Joint CA Aziz TZ. Deep brain stimulation of the pedunculopontine nucleus in Parkinson's disease. Preliminary experience at Oxford. Br J Neurosurg. (2008) 22:S41–4. doi: 10.1080/02688690802448335, PMID: 19085352

[27] Stefani A Lozano AM Peppe A Stanzione P Galati S Tropepi D . Bilateral deep brain stimulation of the pedunculopontine and subthalamic nuclei in severe Parkinson's disease. Brain. (2007) 130:1596–607. doi: 10.1093/brain/awl346 17251240

[28] Thevathasan W Debu B Aziz T Bloem BR Blahak C Butson C . Pedunculopontine nucleus deep brain stimulation in Parkinson's disease: a clinical review. Mov Disord. (2018) 33:10–20. doi: 10.1002/mds.27098 28960543

[29] Baker SN Perez MA. Reticulospinal contributions to gross hand function after human spinal cord injury. J Neurosci. (2017) 37:9778–84. doi: 10.1523/JNEUROSCI.3368-16.2017, PMID: 28871033 PMC5628413

[30] Filli L Engmann AK Zörner B Weinmann O Moraitis T Gullo M . Bridging the gap: a reticulo-propriospinal detour bypassing an incomplete spinal cord injury. J Neurosci. (2014) 34:13399–410. doi: 10.1523/JNEUROSCI.0701-14.2014, PMID: 25274818 PMC6608315

[31] Chang SJ Santamaria AJ Sanchez FJ Villamil LM Saraiva PP Benavides F . Deep brain stimulation of midbrain locomotor circuits in the freely moving pig. Brain Stimul. (2021) 14:467–76. doi: 10.1016/j.brs.2021.02.017, PMID: 33652130 PMC9097921

[32] Mirza Agha B Akbary R Ghasroddashti A Nazari-Ahangarkolaee M Whishaw IQ Mohajerani MH. Cholinergic upregulation by optogenetic stimulation of nucleus basalis after photothrombotic stroke in forelimb somatosensory cortex improves endpoint and motor but not sensory control of skilled reaching in mice. J Cereb Blood Flow Metab. (2021) 41:1608–22. doi: 10.1177/0271678X20968930, PMID: 33103935 PMC8221755

[33] Schuhmann MK Papp L Stoll G Blum R Volkmann J Fluri F. Mesencephalic electrical stimulation reduces Neuroinflammation after Photothrombotic stroke in rats by targeting the cholinergic anti-inflammatory pathway. Int J Mol Sci. (2021) 22:1254. doi: 10.3390/ijms22031254, PMID: 33514001 PMC7865599

[34] Schuhmann MK Stoll G Bohr A Volkmann J Fluri F. Electrical stimulation of the mesencephalic locomotor region attenuates neuronal loss and cytokine expression in the Perifocal region of Photothrombotic stroke in rats. Int J Mol Sci. (2019) 20:2341. doi: 10.3390/ijms20092341, PMID: 31083528 PMC6540310

[35] Zhang S Zhang X Zhong H Li X Wu Y Ju J . Hypothermia evoked by stimulation of medial preoptic nucleus protects the brain in a mouse model of ischaemia. Nat Commun. (2022) 13:6890. doi: 10.1038/s41467-022-34735-2, PMID: 36371436 PMC9653397

[36] Fluri F Malzahn U Homola GA Schuhmann MK Kleinschnitz C Volkmann J. Stimulation of the mesencephalic locomotor region for gait recovery after stroke. Ann Neurol. (2017) 82:828–40. doi: 10.1002/ana.25086, PMID: 29059697

[37] Gopalakrishnan R Cunningham DA Hogue O Schroedel M Campbell BA Plow EB . Cortico-cerebellar connectivity underlying motor control in chronic post-stroke individuals. J Neurosci. (2022) 42:5186–97. doi: 10.1523/JNEUROSCI.2443-21.2022, PMID: 35610051 PMC9236286

[38] Krämer SD Schuhmann MK Schadt F Israel I Samnick S Volkmann J . Changes of cerebral network activity after invasive stimulation of the mesencephalic locomotor region in a rat stroke model. Exp Neurol. (2022a) 347:113884. doi: 10.1016/j.expneurol.2021.113884, PMID: 34624326

[39] Krämer SD Schuhmann MK Volkmann J Fluri F. Deep brain stimulation in the subthalamic nucleus can improve skilled forelimb movements and retune dynamics of striatal networks in a rat stroke model. Int J Mol Sci. (2022b) 23:5862. doi: 10.3390/ijms232415862, PMID: 36555504 PMC9779486

[40] Slow EJ Hamani C Lozano AM Poon YY Moro E. Deep brain stimulation for treatment of dystonia secondary to stroke or trauma. J Neurol Neurosurg Psychiatry. (2015) 86:1046–8. doi: 10.1136/jnnp-2014-308943 25535306

[41] Archer L Snell K Ensor J Hudda MT Collins GS Riley RD. Minimum sample size for external validation of a clinical prediction model with a continuous outcome. Stat Med. (2021) 40:133–46. doi: 10.1002/sim.8766, PMID: 33150684

[42] Ogundimu EO Altman DG Collins GS. Adequate sample size for developing prediction models is not simply related to events per variable. J Clin Epidemiol. (2016) 76:175–82. doi: 10.1016/j.jclinepi.2016.02.031, PMID: 26964707 PMC5045274

[43] Wu D Chen J Zhang X Ilagan R Ding Y Ji X. Selective therapeutic cooling: to maximize benefits and minimize side effects related to hypothermia. J Cereb Blood Flow Metab. (2022) 42:213–5. doi: 10.1177/0271678X211055959, PMID: 34670442 PMC8721772

[44] Liu B Xu J Yang H Yu X Mao Z. PAllidal versus SubThalamic deep brain stimulation for cervical dystonia (PASTS-CD): study protocol for a multicentre randomised controlled trial. BMJ Open. (2023) 13:e073425. doi: 10.1136/bmjopen-2023-073425, PMID: 37832982 PMC10582967

[45] Noga BR Sanchez FJ Villamil LM O’Toole C Kasicki S Olszewski M . LFP oscillations in the mesencephalic locomotor region during voluntary locomotion. Front Neural Circuits. (2017) 11:34. doi: 10.3389/fncir.2017.00034, PMID: 28579945 PMC5437718

[46] Llamas-Ramos R Llamas-Ramos I Pérez-Robledo F Sánchez-González JL Bermejo-Gil BM Frutos-Bernal E . Validity of the telematic Fugl Meyer assessment scale—upper extremity (TFMA-UE) Spanish version. Front Neurol. (2023) 14:1226192. doi: 10.3389/fneur.2023.1226192, PMID: 37638200 PMC10449578

[47] Takebayashi T Takahashi K Amano S Gosho M Sakai M Hashimoto K . Robot-assisted training as self-training for upper-limb hemiplegia in chronic stroke: a randomized controlled trial. Stroke. (2022) 53:2182–91. doi: 10.1161/STROKEAHA.121.037260, PMID: 35345897

[48] Downs S. The berg balance scale. J Physiother. (2015) 61:46. doi: 10.1016/j.jphys.2014.10.002, PMID: 25476663

[49] Miranda-Cantellops N Tiu TK. StatPearls. Treasure Island (FL): StatPearls Publishing (2023).

[50] Downs S Marquez J Chiarelli P. The berg balance scale has high intra- and inter-rater reliability but absolute reliability varies across the scale: a systematic review. J Physiother. (2013) 59:93–9. doi: 10.1016/S1836-9553(13)70161-9, PMID: 23663794

[51] Chen CL Chen CY Chen HC Wu CY Lin KC Hsieh YW . Responsiveness and minimal clinically important difference of modified Ashworth scale in patients with stroke. Eur J Phys Rehabil Med. (2019) 55:754–60. doi: 10.23736/S1973-9087.19.05545-X, PMID: 30868834

[52] Cooper A Musa IM van Deursen R Wiles CM. Electromyography characterization of stretch responses in hemiparetic stroke patients and their relationship with the modified Ashworth scale. Clin Rehabil. (2005) 19:760–6. doi: 10.1191/0269215505cr888oa, PMID: 16250195

[53] Gregson JM Leathley M Moore AP Sharma AK Smith TL Watkins CL. Reliability of the tone assessment scale and the modified Ashworth scale as clinical tools for assessing poststroke spasticity. Arch Phys Med Rehabil. (1999) 80:1013–6. doi: 10.1016/s0003-9993(99)90053-9, PMID: 10489001

[54] Marques-Sule E Arnal-Gómez A Buitrago-Jiménez G Suso-Martí L Cuenca-Martínez F Espí-López GV. Effectiveness of Nintendo Wii and physical therapy in functionality, balance, and daily activities in chronic stroke patients. J Am Med Dir Assoc. (2021) 22:1073–80. doi: 10.1016/j.jamda.2021.01.076, PMID: 33639116

[55] Uysal I Cetisli-Korkmaz N Cavlak U. Assessment of the musculoskeletal performance with squat tests and performance-oriented measurements in older adults. J Back Musculoskelet Rehabil. (2020) 33:735–41. doi: 10.3233/BMR-181283, PMID: 31815685

[56] Howcroft J Lemaire ED Kofman J McIlroy WE. Elderly fall risk prediction using static posturography. PLoS One. (2017) 12:e0172398. doi: 10.1371/journal.pone.0172398, PMID: 28222191 PMC5319679

[57] Veerbeek JM Kwakkel G van Wegen EE Ket JC Heymans MW. Early prediction of outcome of activities of daily living after stroke: a systematic review. Stroke. (2011) 42:1482–8. doi: 10.1161/STROKEAHA.110.604090 21474812

[58] Liu X Zhou M Zhao J Gao Y Wang Y Zhou J . Functional Independence and disability evaluation in stroke patients: optimal cutoff scores for a pictorial-based Longshi scale, Barthel index, and modified Rankin scale. Front Neurol. (2022) 13:710852. doi: 10.3389/fneur.2022.710852, PMID: 35222236 PMC8866832

[59] Zhang Y Xiong Y Yu Q Shen S Chen L Lei X. The activity of daily living (ADL) subgroups and health impairment among Chinese elderly: a latent profile analysis. BMC Geriatr. (2021) 21:30. doi: 10.1186/s12877-020-01986-x, PMID: 33413125 PMC7791986

[60] Abbasi-Ghahramanloo A Soltani-Kermanshahi M Mansori K Khazaei-Pool M Sohrabi M Baradaran HR . Comparison of SF-36 and WHOQoL-BREF in measuring quality of life in patients with type 2 diabetes. Int J Gen Med. (2020) 13:497–506. doi: 10.2147/IJGM.S258953, PMID: 32884330 PMC7434519

[61] Brazier JE Harper R Jones NM O'Cathain A Thomas KJ Usherwood T . Validating the SF-36 health survey questionnaire: new outcome measure for primary care. BMJ. (1992) 305:160–4. doi: 10.1136/bmj.305.6846.160, PMID: 1285753 PMC1883187

[62] Lins L Carvalho FM. SF-36 total score as a single measure of health-related quality of life: scoping review. SAGE Open Med. (2016) 4:205031211667172. doi: 10.1177/2050312116671725 PMC5052926 27757230

[63] Nikolova VL Cleare AJ Young AH Stone JM. Acceptability, tolerability, and estimates of putative treatment effects of probiotics as adjunctive treatment in patients with depression: a randomized clinical trial. JAMA Psychiatry. (2023) 80:842–7. doi: 10.1001/jamapsychiatry.2023.1817, PMID: 37314797 PMC10267847

[64] Liukka M Steven A Vizcaya Moreno MF Sara-aho AM Khakurel J Pearson P . Action after adverse events in healthcare: an integrative literature review. Int J Environ Res Public Health. (2020) 17:717. doi: 10.3390/ijerph17134717, PMID: 32630041 PMC7369881

[65] Rafter N Hickey A Condell S Conroy R O'Connor P Vaughan D . Adverse events in healthcare: learning from mistakes. QJM. (2015) 108:273–7. doi: 10.1093/qjmed/hcu145, PMID: 25078411

[66] Sui Y Tian Y Ko W Wang Z Jia F Horn A . Deep brain stimulation initiative: toward innovative technology, new disease indications, and approaches to current and future clinical challenges in neuromodulation therapy. Front Neurol. (2020) 11:597451. doi: 10.3389/fneur.2020.597451 33584498 PMC7876228

[67] Slotty PJ Poologaindran A Honey CR. A prospective, randomized, blinded assessment of multitarget thalamic and pallidal deep brain stimulation in a case of hemidystonia. Clin Neurol Neurosurg. (2015) 138:16–9. doi: 10.1016/j.clineuro.2015.07.012, PMID: 26241157

[68] Koerbel A Amaral A Zeh HB Wollmann E RFH K Moro C . Treatment of Hemichoreoathetosis with arrhythmic proximal tremor after stroke: the role of zona Incerta as a target for deep brain stimulation. J Mov Disord. (2019) 12:47–51. doi: 10.14802/jmd.18032, PMID: 30732433 PMC6369374

